# The Case of Mr. Ruck

**Published:** 1859-07-01

**Authors:** 


### THE CASE OF MR. RUCK.

It was our intention to have published, *in extenso*, the particulars of this remarkable case, but we are obliged to defer doing so until the next quarter. We cannot, however, permit the present number to go to press without calling the attention of our readers to two or three important matters eliminated during the recent legal proceedings in the Court of

Very important

Queen's Bench. After a trial of three days' duration, the jury (a special one) came to the following verdict:—

"1. That if receiving certain payments as shown made Dr. Conolly part proprietor, they found the fact of receiving the money.

"2. That Dr. Conolly was a regular medical attendant at Moorcroft."

The jury therefore assessed the plaintiff's damages at £500.

"3. That there was not sufficient evidence before them to show that Mr. Barnett, at the time of signing the certificate, was not in actual practice.

"4. That there was not sufficient evidence to show that Dr. Conolly had not examined Mr. Ruck apart from Mr. Barnett."

We have no wish to impugn this decision. If Dr. Conolly was the "regular medical attendant" of the asylum, the verdict was in conformity with the statute law of the land; in other words, the certificates upon which Mr. Ruck was confined were invalid. The questions whether Mr. Ruck was actually insane at the time of his being sent to the asylum, and when Dr. Conolly and Mr. Barnett signed the certificates, and how long his state of lunacy justified his detention there, were matters not referred to or considered by the jury. Medical evidence, however, was adduced in behalf of the plaintiff with the view of establishing that Mr. Ruck ought never to have been certified to be insane, and an attempt was made to prove that he was improperly transferred to and detained in Moorcroft Asylum.

The evidence in support of these allegations was not of a psychological character. It consisted in the opinions of one physician, and three consulting surgeons, all of whom laid no claim to any particular or special knowledge of insanity. The four medical witnesses examined, *viz.*, Dr. Geo. Johnson, Mr. Skey, Mr. Carter, and Mr. Gray *assumed* that Mr. Ruck's case was one of ordinary "*Drunken Insanity*," or *delirium tremens.* Upon this gratuitous assumption and implied hypothesis they deduced their conclusions. There was no evidence that proved the case to be one of *delirium tremens.* No doubt Mr. Ruck indulged freely in the use of stimulants, but this disposition to drink to excess, and his occasional paroxysms of intoxication, were clearly, from the facts deposed to, merely symptoms of a prior and chronic state of disordered mind. The tendency to stimulants sprung out of the insanity being associated with it, and was certainly not its proximate cause.* There was not a tittle of evidence to establish the case to have been as the medical witnesses affirmed, one of *delirium tremens.* It was admitted on both sides that Mr. Ruck laboured under delusions of a dangerous character, and that he was in the habit of occasionally taking stimulants to excess; but in what respect does this condition of disordered mind resemble *delirium tremens?* Mr. Ruck's was not a case of active acute delirium caused by a series of debaucheries, for it was clear that even when entirely free from the influence of intoxicating liquors his mind was under the influence of the same extraordinary delusions respecting his wife's infidelity. However, it suited the purposes of the action to make it appear that Mr. Ruck's case was one of *delirium tremens*, and the medical witnesses *assuming* such to be the fact, were of opinion that there was no justification for his being restrained in a lunatic asylum. No practical physician accustomed to treat cases of *delirium tremens*, and in the habit of distinguishing between this temporary state of mental alienation caused by the presence of toxic agents in the blood, and legitimate attacks of insanity, would have so confounded the two conditions. There is no excuse for so egregious a blunder and so serious a mistake of the evidence adduced at the trial; it constituted the basis upon which the medical men referred to grounded their conclusions. Assume that Dr. Johnson, and Messrs. Skey, Carter, and Gray were right in their diagnosis: when asked whether in their judgment Dr. Conolly and Mr. Barnett were right in considering Mr. Ruck's case to be one of ordinary insanity and transferring it to an asylum—their reply was emphatically *in the negative*, thus passing a severe condemnatory sentence upon the sagacity, knowledge, and humanity of Dr. Conolly and Mr. Barnett.

upon to sit in judgment on a disputed point in practical surgery. We will imagine that Mr. Skey, Mr. Carter, or Mr. John Gray had been called in to deal with a complex and difficult surgical injury. If in twelve or eighteen months afterwards a question were to arise as to whether the surgical case had been treated with scientific skill by any one of these surgeons, and the matter were to be brought into court with a view to obtain compensation for alleged injuries, what would these gentlemen think if Dr. Watson, Dr. Burrows, or Sir James Clark, were to go into the witness-box and swear that these gentlemen had grossly misunderstood the character of the surgical case, and had treated it unskilfully? Would not the profession cry shame upon such evidence? Mr. Skey might with justice say to Sir James Clark and Dr. Watson, what do you know of practical surgery; how is it possible for you physicians to give any opinion on the subject? If you had *seen* the case when I was called in to it, and acquainted yourselves with the great difficulties with which I had to contend in its treatment, you might, perhaps, have been able to guess as to whether I had mismanaged it; but without this knowledge your opinion is not entitled to any consideration, and should not be recorded against a professional brother. If Mr. Ruck entertained, as Dr. Conolly and Mr. Barnett represents at the time of their examination, the most dangerous delusions respecting the fidelity of his wife [against whom the shadow of a suspicion never existed], was he not a fit subject for restraint, and were not Dr. Conolly and Mr. Barnett justified in so certifying? Supposing that Dr. Conolly and Mr. Barnett had hesitated in placing Mr. Ruck under control, and he, influenced by his terrible hallucinations, had sacrificed Mrs. Ruck's life, would not both these medical gentlemen have been seriously censured and called to account for not recognising Mr. Ruck's dangerous insanity, and for not advising that he should be placed in a position of safety and security?

There is one remarkable feature in this case to which we wish to direct particular attention. In reply to a series of questions put to Mr. Ruck by Mr. Montague Chambers, he frankly admitted that the delusions (" suspicions" he termed them) regarding his wife continued on his mind until Mr. Wainwright undertook to examine into their reality. Mr. Wainwright saw Mr. Ruck at the asylum, and assured him *that he had fully investigated into the matter*, and that his, Mr. Ruck's impressions regarding his wife had no foundation in fact. As soon as this statement was made to Mr. Ruck, his delusions, it is alleged, respecting his wife's numerous acts of infidelity immediately vanished. *For ten months previously Mr. Ruck's mind had never apparently been free from these delusions, and in one moment Mr. Wainwright succeeded in dissipating them from his morbid imagination.* "*Veni Vidi, Vici*," exclaims this wonderful psychological lawyer. Let not the question be asked, "*Who can minister to a mind diseased?*" as long as Mr. Wainwright's valuable services can, be obtained for the benefit of the insane.

Considering this matter in its strictly legal bearing, perhaps the jury had no alternative but to find a verdict for the plaintiff; but were not the damages (£500) *excessive*, and is there no hope of appealing against the verdict on this ground? Was it not necessary for the safety of human life that Mr. Ruck should be confined as a dangerous lunatic, and if so, ought not that slight irregularity in Dr. Conolly's certificate to be considered with a lenient eye?

The editor of a weekly paper, when alluding to this case, justly remarks, "We strongly protest against the doctrine that no restrictive measures are ever to be employed until an insane person takes to knife, poker, or razor, and proves his madness by slaying somebody else. It is by well-timed and kindly precautionary measures that real madness may be averted, and the patient restored to health; and it is by no means improbable that the gentleman who was plaintiff in this case, owes his ability to be so to the period of restraint to which he appears to have been somewhat irregularly confined."* The following remarks on the case are important, as proceeding from the *Solicitor's Journal.*

"The prominent feature in the Courts during the week has been the case of *Ruck* v. *Stilwell*, an action for damages for illegal detention in a lunatic asylum. It appears that the certificate authorizing the detention of the plaintiff was signed by Dr. Conolly and a Mr. Barnett; and it was contended by the plaintiff that the latter was incapacitated, because he had ceased to practise, and that the former was disqualified, because he had a pecuniary interest in the establishment to which Mr. Ruck was sent. The jury thought that the case, as related to Mr. Barnett, was not made out, there being no evidence that he was not practising as a medical man; but with regard to Dr. Conolly, they found that he was the regular professional attendant at the asylum, and, therefore, unable (under the statute) to sign the certificate. The plaintiff accordingly obtained a verdict, and damages to the amount of £500, but subject to the opinion of the Court above on a point reserved. We have no hesitation in expressing our opinion, that the pecuniary mulct inflicted on Dr. Stilwell is unjust, because no one can doubt that the plaintiff was insane at the time of his confinement, and that he benefited by the restraint, although there may have been some irregularity in the signing the certificate. That irregularity was a subject for fair comment, and, slight as it was, it has been made the most of for Mr. Ruck."

* *The Era.*

